# The Role of Registries in Neurotrauma Research: Translating Data Into Health Policy That Enhances Patient Care: Comment on "Neurotrauma Surveillance in National Registries of Low- and Middle-Income Countries: A Scoping Review and Comparative Analysis of Data Dictionaries"

**DOI:** 10.34172/ijhpm.2023.7521

**Published:** 2023-02-26

**Authors:** Nqobile Thango, Ronnie E. Baticulon, Laura Lippa

**Affiliations:** ^1^Division of Neurosurgery, Department of Surgery, University of Cape Town, Cape Town, South Africa.; ^2^Neuroscience Institute, University of Cape Town, Cape Town, South Africa.; ^3^Division of Neurosurgery, Philippine General Hospital, University of the Philippines Manila, Manila, Philippines.; ^4^Department of Neurosurgery, ASST Ospedale Maggiore Niguarda, Milano, Italy.

**Keywords:** TBI, Health Policy, Neurotrauma Registry, Neurotrauma Surveillance, National Data Registries, Research

## Abstract

The paucity of robust neurotrauma data is felt most in regions that experience a higher burden of traumatic brain injury (TBI). The scoping review done by Barthélemy et al provides insight into the current state of national registries in low- and middle-income countries (LMICs) while also exploring the tools required to standardize data collection. In this commentary, we reflect on the barriers to data collection (ie, creation and maintenance of a TBI registry) and explore how registries can aid both scientific output and preventative public awareness campaigns that may pave the way to improved health policy and social change that avert mortality and morbidity from TBI.

 Traumatic brain injury (TBI) is a major public health concern. The medical treatment of the injury, its many sequelae, and the resulting neurological disability of survivors result in a significant socioeconomic burden on healthcare systems globally. In addition, 89% of worldwide TBI mortality occurs in young adults within low- and middle-income countries (LMICs).^1^ In the pediatric population, the long-term consequences are often more devastating due to their age and developmental potential. In resource-constrained environments, many TBIs go untreated; the severe ones often do not make it to specialized neurosurgical centres. Thus the data remains uncaptured in national statistics, and the extent of this pandemic is hidden from national health ministries and under-represented in healthcare policies. This cascade of events results in human suffering and economic loss.

 In this commentary, we reflect on the barriers to data collection (ie, creation and maintenance of a TBI registry) and explore how registries can aid both scientific output and preventative public awareness campaigns that may pave the way to improved health policy and social change that avert mortality and morbidity from TBI.

 While the monumental modeling work of the Global Burden of Disease series provides general trends, in the 2016 edition reported sub-Saharian African neurotrauma rates were lower than European ones: we know this to be flawed conclusion, most likely driven by huge data gaps.

 Barthelémy et al describe the root of this very well^2^: while patient registries aim to improve patient care and assist in the surveillance of disease burdens and clinical research, only 16 LMICs Countries hold a national trauma registry, all covering most (but only one covering entirely) the World Health Organization-International Registry for Trauma and Emergency Care (WHO-IRTEC) minimum dataset for injury.

 According to the WHO, registry criteria (version 2.1, April 2009) for data to be collected in a standardized manner, it should meet the standards of the International Clinical Trials Registry. This ensures that the research is accessible to all involved in healthcare decision-making.

 Within the medical community, the concept of patient registries is not new: information gained from observational research gives vital information which can assist in improving clinical guidelines, and neurosurgeons are often the promoters of evidence-based clinical practice.^2-5^

 The cruciality of clinical research is easily grasped, as it gives insight into the disease burden within a population group and improves patient care, however considerable gaps exist between epidemiology and research output.^6^ Despite the massive clinical experience our colleagues accumulate, trauma literature suffers from an overall limited research output from LMICs: while the greatest burden of neurosurgical diseases is found in LMICs, they also suffer from a critical shortage of neurosurgical workforce.

 Neurosurgeons, specifically in Africa and South-East Asia, are in the unique position of providing essential services to a large portion of their population.^4^ This translates into a vast experience in TBI management, and as such there is great need to learn from their real-world expertise. However, the massive surgical burden (both met and unmet) derived from understaffing may prevent our colleagues in LMICs from engaging in formal clinical research, as busy clinical practices may not be compatible with academic endeavours.^5^

 Clinical duties are not the only factor limiting research capacity and output: a wider lack of resources (eg, research workforce, research formation, reliable funding, dedicated personnel) and lack of perception are all contextual factors that hinder efforts. In fact, we can consider them barriers in all aspect of public health, from surveillance^7^ to implementation of measures. Shumba and Lusambili^8^ have examined the many barriers to research capacity such as language barriers, social disparities, dedicated fundings, dissemination barriers, perception of the importance of a systemic approach to research capacity building. When implementation occurs, results are promising and readily perceivable: in a review of trauma reports performed at Mbarara Regional Referral Hospital, Uganda^9^ on the period prior and following the introduction of a trauma registry with electronic patient registration system, results showed an 20.9 fold increase for completed trauma patient documentation. The clinical and research potential implications of such implementation are considerable – documentation, follow up of patients, preventative efforts based on epidemiology – and context-informed results can trigger a virtuous circle of self sustainance and targeted education: the Authors highlight that results can leverage for increased government funding and subsequent long-term registry sustainability.

 Along with the awareness of the current weight of geographical location on TBI clinical outcomes comes that of the intrinsic characteristic of preventability which we must act on to modify adverse patient outcomes. Very little pathology has the ubiquitariety and incidence of neurotrauma, while carrying such a potential for improvement.

 Neurosurgeons can further contribute to evidence-informed policy-making, bringing their expertise to preventative approaches and protocols. In a review about neurotrauma and randomized controlled trials preventions regarding both high-income countries (HICs) and LMICs^10^ including over 400 articles (the majority coming from HICs) the most common preventative approach was legislation/policy, followed by helmet use, in both HICs and LMICs. The most evident discrepancies were in the use of technology and in prehospital care, which were lacking in LMICs. Primary (education, legislation, awareness, envoronmental engineering, protective equipment) and secondary (emergency medical services and prehospital care) prevention started at a broader level, whilst tertiary prevention (rehabilitation) was mostly implemented in smaller community settings.

 Following Kotter’s theory of change,^11^ in order to create change, we need to constantly generate short-term wins to justify efforts and motivate further committment. Very little can generate a motivational cascade as appreciating the results of healthcare policies.

 Policy-making is “an inherently political process involving competing calls for the attention and for the finite resources available.”^12^ Clinicians can have a pivotal role in the development of National Surgical Obstetric and Anesthesia Plans or comprehensive policy recommendations, such as the ones on head and spine injury care in LMICs issued in 2013: here, Authors merged a policy-oriented methodology from the National Surgical Obstetric and Anesthesia Plan framework with existing trauma guidelines. Employing the same framework already in place simplifies the integration of the recommendations, helping *de facto *a clinical commitment.

 While it is always possibile to see an important policy proposal rejected in favour to other interests, it is mandatory to provide every sound evidence in order to back-up its prioritization and subsequent adoption and enforcement. Evidence alone, however, does not always do the trick: we have to reorient power relations to uptake evidence and drive significant change in healthcare.^13,14^

 For most clinicians, participation in global governmental events such as the World Health Assembly might be a step out of the comfort zone15 but it can lead to meaningful sharing of knowledge and, ultimately, communication efforts eventually translate into ground-breaking Resolutions (like 68.31 and 68.15) that are pathfinders for improvement.

 We commend Barthélemy et al^2^ for their effort; the need for data inclusion to be published in peer-reviewed journals is a declaration of their commitment to surveillance and continued resource allocation, leading to healthcare equity. It is our hope that future studies may shed further light on the specific uses of such registries and offer practical evidence of the efficacy to promote their usage in countries who may not currently have active TBI registries. The Authors aimed to enhance the quality of the results of this scoping review by having a narrow inclusion criterion while utilizing non-randomized sampling, namely cold contact, and convenience sampling. Although the use of convenience sampling may overcome the limitations of some research methods, it carries the risk of selection bias. In addition, the risk of low response rates from cold contacts may leave out essential information that may be useful to Ministry of Health.

 As 2030 rapidly approaches despite all the many tentatives the human race is trying to put up to end itself, the objectives for Sustainable Development Goal 3.6 call us to be transformative, to “challenge orthodoxies” and grab this opportunity we have to coalesce and shield: investing in collaborative health research is the only way forward for a true greater good.

## Acknowledgements

 We wish to dedicate this commentary to the memory of Prof. Paul Farmer, whose integrity and light will guide us and generations to come.

## Ethical issues

 Not applicable.

## Competing interests

 Authors declare that they have no competing interests.

## Authors’ contributions

 All three authors contributed to all process of conception-research-writing of the paper.
